# Conditions of the Erie County Poor House*From the Commercial Advertiser of July 20th.

**Published:** 1854-08

**Authors:** 


*Condition of the Erie County Poor House.**—Painful rumors have been in circulation for a day or two, relative to the mortality at the Poor House, and its causes. To investigate the truth of these rumors, the Board of Health, accompanied by several physicans, visited the premises, situated five miles north of the city, on the evening of the 19th, inst. Whatever we may have to say of the state of things there, is derived from facts ascertained at this visit, and however incredible our story may appear, we assure our readers, that it is but too truthful.

The Board found that, during the preceding twenty-four hours, 15 insane persons had died of the cholera; and that four more were then in collapse, and could live but a few hours. This makes a total of 19 deaths in some 36 hours, from a house containing some 53 inmates. All these deaths occurred from a building isolated from the main edifice, in which also cholera was prevalent, though not with such an unparalleled fatality.

This great number of deaths, occurring in a single building, and exceeding the total of mortality in the remainder of a city of 80,000 inhabitants must have a local cause within the control of its managers. We have a right to assume that there is blame somewhere.

Uufortunately the investigations of the Board of Health render it too plain that, aside from insufficient ventilation and other causes incident to the construction of the building, the manifest cause of this mortality is in the diet of the house—a diet which exceeds, in its accurate estimate of the starvation point, anything which Dickens ever described in the a work-house, or Dotheboy's Hall.

The Board heard but one story from the employees in the house, and we cannot better detail the actual condition of the patients, than by giving a resume of the daily fare, its quantity and quality:

Breakfast—A piece of bread about 5 inches square by ⅔ of an inch thick, a little salt pork with *coffee*, made from barley, and sweetened with the cheapest of molasses.

Dinner—Same as breakfast, minus the coffee. Supper—Bread and tea. Once a week mutton soup.

As to quality, the nurse in charge of the children said that the bread was never sufficiently baked, and was frequently so sour as to curdle milk. On rolling it in the hand it worked up into dough very readily. No butter is given on the bread.

---

* From the Commercial Advertiser of July 20th.

The pork was rusty, and slippery necks and shoulders.

For a few days back they had had some mutton served out. The ration for the day was seen; about two ounces (bone and all) being given to each.

Vegetables—During three or four months of winter and spring none were given out. This was owing to the high price of potatoes, but no substitutes, such as beets, carrots or turnips were provided. Since the appearance of cholera, no vegetable of any kind. As a consequence of this beautiful management there has been one death from scurvy, (a disease hardly ever known except in ships on long voyages,) and four other cases are now in the house.

Hospital diet is the same as house diet. After a great deal of argument and remonstrance from the attending and house physican, they have obtained from the liberal Superintendent in charge an occasional ration of beef broth for the sick.—Its mode of manufacture may interest our lady readers. The Superintendent purchases, from the refuge from the slaughter-houses, certain shanks of beef at 12½ cents apiece. He usually buys one at a time; in moments of extravagance and disregard for the County Treasury, two. One of these shanks is boiled in water enough to give thirty odd patients a pint of broth each. When two shanks are boiled, twice the quantity of water is added, and half is religiously saved till a second day.

The nursing is done by a sore-legged Irish pauper, who takes especial care of his fellow countrymen, at the expense of French or American paupers.

We intended to speak of the ventilation, but we are sick of the subject. Suffice it to say that the ground floor is on a level with the earth, paved with bricks, laid directly upon the clap; that all the dining, cook and wash rooms, are on this floor; and that the steam and stench of all the household offices find vent only through the joints of the floors above—none of the ceilings are lathed and plastered; and the foul odors of the basement ascend to the fourth story. There is not a hall in the building; and the ventilators connecting with the flues are perfect shams. Throughout the whole house there is a sickening, decaying odor. All these remarks apply to both buildings alike.

Thus far we have confined ourselves to a naked statement of the plain, disgusting facts. But we have a word by way of improvment.

Here are more than three hundred paupers kept in a foul, ill-ventilated building, on a diet which will just keep the wheels of life in motion.

For month after month the thing has gone on, producing its legitimate and murderous results. Lying-in women have died of fever, leaving their children, and the numerous foundlings brought to the house, to die of starvation together. Brought there as bright, healthy infants, these little offsprings of misfortune and crime have scarcely an average of four weeks of life in this great lazar house. Older children become idiotic, dwarfed, inanimate; and men and women die from a hundred diseases, written down upon the case-book with Latin names, but which might be better called starvation—starvation of God's air and the fruit of God's earth. The present mortality from cholera is only a signal manifestation of what has been going on for months; a small addition to the daily deaths; a change in the particular mode of dying; or to look on it in the most favorable light, a substitution of death for imbecility; or rescue from a life which is worse than death.

We have no word of censure for the Superintendent in charge. We leave the facts with the people. Let them decide whether a miserable economy; a small poor tax at the close of the year, is any justification of the facts we have related—facts which can be sustained by the names of those whom the community know and can trust.

For one thing we are thankful—we have a board of Health which we believe will do its duty, and, so far as in it lies, reform this great abuse—this plague spot on the body politic.

---

"*A Protocol to Critics.*"—In the New Orleans Journal for July, immediately following a most caustic* review of "Woman, her Diseases, and her Remedies," is a letter from Dr. Meigs to the Editor, wherein he denounces the "young gentlemen, the sophomore *scollards* who assail" him, for their unmannerly criticisms. As a warning to these "young gentlemen," among whom he must now include the veteran Dowler, he relates the pathetic story of the prophet Elisha, who met sundry naughty boys who said, "go up, go up, thou bald-head!" when the Lord sent two she bears out of the mountain, and "they tare forty-and-two of those children that day."

What a lesson to Young America and Young Physic!

We only allude to this letter because it is one of the curiosities of medical literature. Dr. Meigs has one, and only one, answer to the critics who attack his books. Like Pindar's razors, they *sell!* But it is a private opinion of

* We err in saying "*most* caustic," According to Dr. Meigs, the "caustic crayon" has three methods of application, the "indifferent, the antiphlogistic, and the destructive." Dr. Dowler evidently intends only an "antiphlogistic contact."

our own, that the tribe of critics have contributed largely to the pecuniary success of these literary labors. The spicy quotations always given, stimulate the appetite of the reader for the whole book. Seriously we do not see wherein Dr. Meigs has been injured or unfairly used. H.

